# Prompt Architecture as a High-Impact Design Factor in Expert-Rated Clinical Documentation Quality: A Controlled Comparative Study in Inpatient Rehabilitation

**DOI:** 10.3390/bioengineering13060617

**Published:** 2026-05-25

**Authors:** Idoia Eceizabarrena-Matxinandiarena, Emilio Javier Frutos-Reoyo, José Ignacio Guerrero-Rojas, Clara Vidal-Millet, Pedro Ignacio Tejada-Ezquerro, Elena Roldan-Arcelus, Irene de Torres-García, Judith Sanchez-Raya, Lourdes Gil-Fraguas, María Hernandez-Manada, Carolina de Miguel-Benadiba, Josep Maria Monguet i Fierro, Alex Trejo Omeñaca, Michelle Cavariani Catta-Preta, Astrid Teixeira-Taborda, Natalia Álvarez-Bandrés, Raquel Cutillas-Ruiz, Helena Bascuñana-Ambrós

**Affiliations:** 1 Department of Physical Medicine and Rehabilitation, Hospital Universitario Donostia, 20014 San Sebastian, Spain; 2 School of Medicine, University of the Basque Country (EHU), 48940 Leioa, Spain; 3 Spanish Society of Physical Medicine and Rehabilitation (SERMEF), 28016 Madrid, Spain; efrutosr@saludcastillayleon.es (E.J.F.-R.); jigr05@sescam.jccm.es (J.I.G.-R.); vidal_clamil@gva.es (C.V.-M.); pedroignacio.tejadaezquerro@osakidetza.eus (P.I.T.-E.); le.roldan.arcelus@navarra.es (E.R.-A.); irene.torres.sspa@juntadeandalucia.es (I.d.T.-G.); judith.sanchez@vallhebron.cat (J.S.-R.); lourdesgilfraguas@yahoo.es (L.G.-F.); maria.hernandez.manada@sermef.es (M.H.-M.); carolina.demiguel.benadiba@sermef.es (C.d.M.-B.); josep@innex.io (J.M.M.i.F.); astrid.teixeira.taborda@sermef.es (A.T.-T.); nalvarez@riojasalud.es (N.Á.-B.); rcutillas@fjd.es (R.C.-R.); hbascunana@santpau.cat (H.B.-A.); 4 Department of Physical Medicine and Rehabilitation, Hospital Universitario Río Hortega de Valladolid, 47012 Valladolid, Spain; 5 Department of Physical Medicine and Rehabilitation, Hospital General de Segovia, 40002 Segovia, Spain; 6 Department of Physical Medicine and Rehabilitation, Hospital General Universitario La Mancha Centro, 13600 Alcázar de San Juan, Spain; 7 Department of Physical Medicine and Rehabilitation, Hospital Universitario de la Ribera, 46600 Alzira, Spain; 8 Department of Physical Medicine and Rehabilitation, Hospital de Gorliz, 48630 Gorliz, Spain; 9 Department of Physical Medicine and Rehabilitation, Hospital Universitario de Navarra, 31004 Pamplona, Spain; 10 Department of Physical Medicine and Rehabilitation, Hospital Universitario Reina Sofía, 14004 Córdoba, Spain; 11 Department of Physical Medicine and Rehabilitation, Hospital Universitari Vall d’Hebron, 08035 Barcelona, Spain; 12 Department of Physical Medicine and Rehabilitation, Hospital Universitario de Guadalajara, 19002 Guadalajara, Spain; 13 Khore Global Consulting, 28043 Madrid, Spain; 14 Department of Physical Medicine and Rehabilitation, Hospital Beata María Ana, 28007 Madrid, Spain; 15 Department of Engineering Graphics and Design, Universitat Politècnica de Catalunya (UPC), 08028 Barcelona, Spain; alex@innex.io; 16 Innex Labs, 08800 Vilanova i la Geltrú, Spain; michelle@innex.io; 17 Department of Physical Medicine and Rehabilitation, Hospital Universitario Fundación Jiménez Díaz, 28040 Madrid, Spain; 18 Department of Physical Medicine and Rehabilitation, Hospital Universitario San Pedro, 26006 Logroño, Spain; 19 Department of Physical Medicine and Rehabilitation, Hospital de la Santa Creu i Sant Pau, Campus Salut, 08041 Barcelona, Spain

**Keywords:** artificial intelligence, clinical documentation, discharge reports, large language models, medical writing, prompt architecture, prompt engineering, rehabilitation medicine

## Abstract

Large language models (LLMs) are being increasingly explored to support clinical documentation, yet the influence of prompting architecture on documentation quality in complex longitudinal contexts remains insufficiently characterized. This controlled retrospective methodological study evaluated three prompting strategies—single prompt (SP), section-based prompt (SBP), and section-based prompt with writing refinement (SBP+W)—for generating inpatient rehabilitation discharge reports using OpenAI large language model (GPT-5.2). Twenty anonymized inpatient rehabilitation cases involving prolonged hospital stays and multidimensional functional documentation were processed under standardized model conditions. AI-generated reports were compared with human-authored summaries. Two blinded board-certified rehabilitation physicians independently evaluated outputs using a structured four-point ordinal scale assessing structural integrity, clinical coherence, completeness, and readability. Inter-rater reliability was estimated with quadratic weighted Cohen’s kappa and bootstrap confidence intervals. Group differences were analyzed using non-parametric testing and exploratory multivariable modeling. All LLM prompting strategies achieved significantly higher expert-rated quality scores than human-authored reports (*p* < 0.025). SBP demonstrated the highest median performance and strongest regression effect, although differences among LLM-based strategies were not statistically significant after correction. Prompting strategies explained more variability in expert ratings than case-level factors. Structured section-based prompting may represent a practical design lever for improving perceived quality in AI-assisted clinical documentation workflows. Larger prospective studies are needed to evaluate reliability, safety, clinical utility, and implementation in real-world workflows.

## 1. Introduction

Large language models (LLMs) have rapidly emerged as powerful generative systems capable of producing structured natural language across a wide range of knowledge-intensive domains, including healthcare. Recent studies demonstrate that LLM-based systems can assist clinicians in producing clinical documentation, discharge summaries, and patient instructions with levels of readability and structural consistency comparable to, and in some contexts exceeding, those of human-authored documentation [1,2,3,4,5,6]. Given that clinical documentation represents a major source of administrative workload for physicians, AI-assisted documentation tools are increasingly explored as a strategy to improve efficiency, reduce cognitive burden, and enhance documentation standardization.

Despite these advances, the reliability of LLM-generated clinical documentation remains a central concern. Prior research has highlighted several limitations, including hallucinated information, omission of clinically relevant details, contextual drift in long document settings, and instability in reasoning when synthesizing heterogeneous medical information [7,8,9,10]. These challenges are particularly pronounced in longitudinal clinical contexts, where documentation must integrate multiple assessments, evolving diagnoses, and multidisciplinary interventions into a coherent narrative. Inpatient rehabilitation discharge summaries represent a demanding example of such documentation tasks. It requires synthesis of functional assessments, therapeutic interventions, and recovery trajectories within a structured clinical report.

From a systems design perspective, increasing attention has focused on prompt engineering as a key factor influencing LLM behavior. Prompt architecture, define as the structural design of instructions provided to the model, can shape how generative models interpret tasks, organize information, and produce outputs. Contemporary prompt engineering frameworks describe multiple architectural strategies including monolithic prompts, modular task decomposition, hierarchical prompting, and chain-of-thought reasoning [11,12,13,14,15,16]. Experimental work in computational domains suggests that modular and decomposed prompting strategies may improve reasoning stability, reduce contextual interference, and enhance output consistency.

However, empirical evidence evaluating the impact of prompt architecture in real-world clinical documentation tasks remains limited. Most existing healthcare studies focus on evaluating overall output quality or safety of AI-generated documentation without systematically isolating prompt structure as an experimental variable [3,4,17,18]. As a result, it remains unclear whether observed variability in LLM-generated documentation primarily reflects intrinsic model properties (e.g., model scale or training data) or design choices in prompt architecture.

Understanding the relative influence of prompt architecture is important for the safe implementation of LLM systems in clinical workflows. If prompt design significantly affects documentation quality, improvements in reliability could be realized by implementing controllable interventions at the interface level rather than requiring new model training or scaling. Such insights would position prompt architecture as a practical systems engineering lever for optimizing generative AI in healthcare environments.

To address this gap, we conducted a controlled comparative methodological study evaluating three prompting architectures—single prompt (SP), section-based prompt (SBP), and section-based prompt with writing refinement (SBP+W)—for the generation of inpatient rehabilitation discharge reports using an OpenAI large language model (GPT-5.2). We hypothesized that modular section-based prompting would improve expert-rated documentation quality compared with monolithic prompting approaches, and that prompt architecture would explain a substantial proportion of variability in documentation performance across clinical cases.

## 2. Materials and Methods

### 2.1. Study Design

We conducted a retrospective, comparative methodological study to evaluate the impact of different prompting architectures on the quality of AI-generated rehabilitation discharge reports. The study was designed following methodological principles commonly applied in clinical artificial intelligence evaluation frameworks, including controlled input conditions, blinded expert assessment, and structured qualitative and quantitative analysis.

The primary objective was to compare three prompting strategies in terms of structured clinical documentation quality, functional coherence, and overall expert-rated performance.

**Figure 1:**
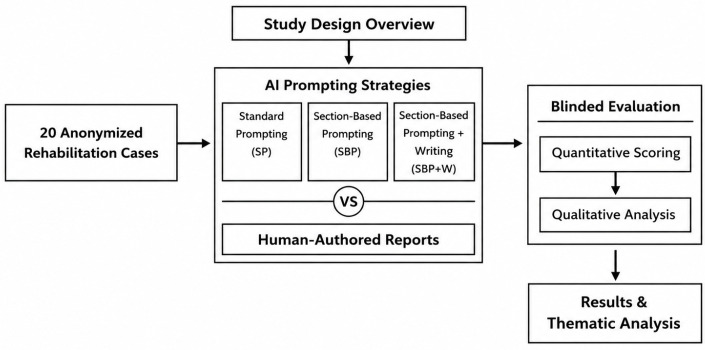
Schematic representation of the study design. Twenty anonymized inpatient rehabilitation cases were processed using three AI prompting strategies (SP, SBP, SBP+W) and compared with human-authored reports. Two board-certified rehabilitation physicians evaluated all outputs under blinded conditions. Quantitative ordinal scoring and qualitative affinity-based thematic analysis were performed.

Figure 1 shows a detailed schematic representation of the study.

### 2.2. Data Source and Case Selection

Twenty anonymized inpatient rehabilitation cases were retrospectively selected from multiple national public hospitals. Cases were purposely sampled to reflect the complexity of rehabilitation medicine, including prolonged length of stay, multidimensional functional assessments, and extensive narrative documentation. De-identified clinical data was utilized prior to model processing, in compliance with all relevant data-protection standards. No identifiable patient information was introduced into the generative system.

### 2.3. Prompt Design Framework and Cognitive Constraints

The three prompt architectures were designed using the Prompt Canvas framework [19], which defines instruction development across ten elements: role, audience, context, task, process, anchors, format, style, references, and evaluation. This framework was selected to ensure methodological transparency, reproducibility, and clinical safety. Representative prompt excerpts and architecture-specific operational constraints are provided in Appendix B to improve methodological reproducibility while preserving non-confidential implementation details.

The design process addressed three key constraints associated with longitudinal clinical data:

1. Context window limitations: Long-term rehabilitation records generate large input volumes that may approach the model’s effective context capacity, resulting in attention decay (i.e., “Lost in the Middle”), where instructions lose relative influence.
2. Cognitive load: High-density clinical documentation, including repetitive entries and multiple interdepartmental transitions, increases processing complexity and may negatively affect output quality.
3. Traceability and safety: Clinical reports require strict alignment with source data. Accordingly, hallucination mitigation and auditability were treated as primary design criteria.

### 2.4. Prompt Architectures

#### 2.4.1. Unified Instruction Architecture

The unified architecture processes the complete clinical context and instructions within a single inference step. Internally, it follows four sequential stages: analysis, synthesis, report generation, and validation. The prompt included fixed role assignment (“specialist in Physical and Rehabilitation Medicine”), explicit hallucination prevention constraints (“Do not invent findings, doses, dates, or treatments”), chronological prioritization rules, and mandatory reporting of missing information when source data are incomplete.

Safety is implemented through uncertainty detection mechanisms that prompt the model to explicitly identify missing or inconsistent data. This approach preserves global contextual coherence but does not enforce explicit traceability.

The main limitation of this pattern is that performance may decrease with increasing input size due to cognitive overload and reduced constraint effectiveness.

#### 2.4.2. Segmented Instruction Architecture

The segmented architecture decomposes the task into ten predefined report sections (e.g., clinical history, physical examination, diagnosis), each processed independently. Each prompt executes two steps: (i) extraction of relevant data and (ii) structured narrative generation. Each section-specific instruction contained standardized exclusion rules, temporal validation constraints, and internal self-review loops requiring the model to cross-check generated recommendations against the source documentation before output finalization.

To ensure consistency, a mandatory internal validation phase cross-references generated outputs with source data before finalization. While this mechanism minimizes cognitive load and enhances local accuracy, it may inadvertently compromise global coherence across broader sections.

#### 2.4.3. Segmented Architecture with Sequential Writing Refinement

This architecture extends segmentation by separating extraction and generation into independent stages (20 prompts in total), following a separation of concerns approach.

During the extraction phase, outputs were constrained to structured formats (e.g., JSON) with explicit source attribution:

*“sources”: [{“field”: “field_name”, “text_snippet”: “…”}]*

The extraction prompts prohibited free narrative generation and required deterministic mapping between extracted clinical variables and source text fragments. During the refinement stage, prompts applied linguistic normalization constraints while explicitly prohibiting incorporation of novel clinical content.

Output generation was constrained by formal linguistic parameters to avoid data alteration and maintain high-fidelity traceability. These restrictions, however, require a more elaborate implementation framework and limit narrative adaptability. Table 1 presents a summary of the key features of the evaluated prompt architectures, and Table 2 details how the Prompt Canvas framework was applied in their design.

### 2.5. Model Configuration and Execution

We performed all generations using GPT-5.2 via web access, using standard time thinking mode and default model parameters. The model uses advanced reasoning capacity and improved instruction adherence relative to smaller-scale models. Prompt templates, structural constraints, and section definitions remained fixed across all cases. No iterative prompt optimization or case-specific tuning was performed after protocol finalization.

To ensure methodological rigor and reproducibility:

- Identical system-level instructions were maintained across all conditions.
- Generation parameters (web access, GPT-5.2; default parameters, standard reasoning mode) were kept fixed in all cases.
- No case-specific prompt modifications were introduced once the experimental protocol was finalized.
- Each case was processed independently to prevent cross-case contextual contamination.

### 2.6. Outcome Measures

#### 2.6.1. Quantitative Expert Evaluation

For each clinical case, four discharge reports were evaluated:

- A: Original human-authored report.
- B: SP-generated report.
- C: SBP-generated report.
- D: SBP+W-generated report.

Two independent board-certified rehabilitation physicians assessed all reports under blinded conditions with regard to the generation strategy.

Reports were rated using a four-point ordinal scale assessing structural integrity, clinical accuracy, coherence, completeness, and readability (see Appendix A for more details):

- Poor = 0.
- Fair = 1.
- Good = 2.
- Excellent = 3.

In cases where inter-rater discrepancies exceeded two points, a third independent expert provided an additional evaluation. Rather than assigning the final score to the third evaluator, all available ratings were considered, and the median of the three evaluations was considered final for analysis.

Inter-rater reliability was assessed using weighted Cohen’s kappa.

#### 2.6.2. Qualitative Expert Feedback

Reviewers provided free-text comments regarding the strengths and weaknesses of each report. These qualitative data underwent structured thematic analysis.

An affinity diagram methodology was applied to cluster qualitative observations into emergent thematic categories (e.g., coherence, functional specificity, redundancy, clinical reasoning adequacy, stylistic clarity). Two researchers independently coded comments, followed by consensus-based category consolidation to enhance analytic rigor.

#### 2.6.3. Exploratory Hallucination Analysis Description

An exploratory phrase-level hallucination audit was conducted to assess the factual consistency of generated clinical reports. A total of 10 reports were randomly selected, including AI-generated outputs (across different prompting strategies) with human-authored reports as a comparator.

Each report was segmented into individual phrases, which were independently classified into four predefined categories:

- No hallucination: Information was explicitly supported by the source clinical progress notes.
- Deduction: Information was not explicitly stated but reasonably inferable by a clinician.
- Hallucination: Information was not supported by the notes nor inferable, or it was clearly incorrect.
- Title: Non-clinical content (e.g., headings or structural text), which was excluded from analysis.

The primary analysis included only clinically relevant phrases (i.e., excluding titles). The audit was conducted at the phrase level, and summary metrics were calculated both globally and stratified by generation strategy. Additionally, a report-level hallucination rate was computed for each document, and median values were reported per strategy.

### 2.7. Statistical Analysis

We conducted all analyses using validated statistical software. Given the ordinal nature of expert ratings (four-point scale, 0–3), we applied non-parametric methods for primary comparisons.

We summarized data as mean ± standard deviation (SD) and median with interquartile range (IQR).

We compared documentation quality across the four report types (human-authored, SP, SBP, and SBP+W) using the Friedman test for repeated measures. We estimated size effect with Kendall’s W.

When the Friedman test indicated significance, we performed post hoc pairwise comparisons using Wilcoxon signed-rank tests with Bonferroni correction.

We assessed the relative contribution of prompting strategy and case-level variability using a multivariable linear regression model, with expert rating as the dependent variable and case and strategy as predictor. We defined the human-authored report served as the reference category. We evaluated model performance using R^2^, adjusted R^2^, F-statistics, and corresponding *p*-values.

We assessed inter-rater reliability using quadratic weighted Cohen’s kappa (κ) and calculated 95% confidence intervals using bootstrap resampling (2000 iterations).

All tests were two-tailed, and we set statistical significance at *p* < 0.05.

All statistical analyses were conducted using RStudio 2026.01.1 Build 403 (Posit Software, PBC, Boston, MA, USA) running R 4.5.3 (R Foundation for Statistical Computing, Vienna, Austria). ChatGPT (GPT-5.2; OpenAI, San Francisco, CA, USA) was employed for qualitative analysis and research intervention support using standard reasoning mode with default parameters.

### 2.8. Ethical Considerations

We conducted this study using fully anonymized retrospective clinical data. No patient contact or intervention occurred. Ethical review was not required due to the exclusive use of de-identified data and the non-interventional design.

### 2.9. Use of Generative Artificial Intelligence

Generative artificial intelligence (GPT-5.2, web version; default parameters, standard reasoning mode) was the primary object of evaluation in this methodological study. We used the model exclusively to generate discharge reports under controlled prompting conditions. We did not make any automated clinical decisions. Human experts evaluated all outputs. The authors supervised prompt design, analytical procedures, and manuscript preparation, and assumed full responsibility for the integrity and accuracy of the study.

## 3. Results

### 3.1. Study Sample

Twenty inpatient rehabilitation cases were included (see descriptive statistics in Table 3). For each case, we generated four discharge reports (human-authored, SP, SBP, and SBP+W), resulting in 80 evaluated documents. The cases showed prolonged admissions and complex longitudinal documentation.

### 3.2. Comparative Performance Across Prompting Strategies

**Figure 2:**
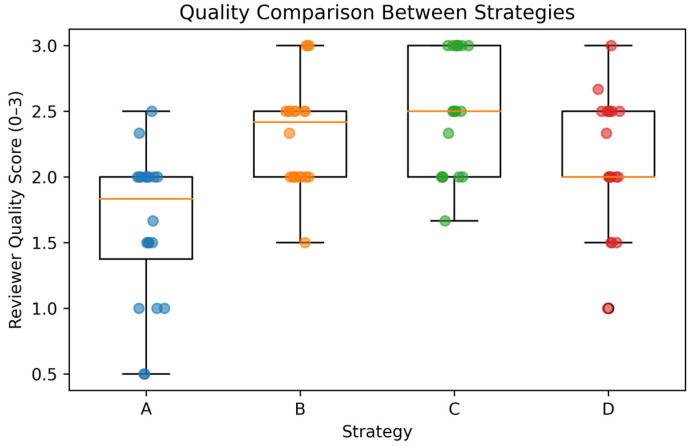
Distribution of reviewer quality scores across strategies. Boxplots represent median and interquartile range of averaged reviewer scores for each strategy.

A Friedman test revealed a statistically significant difference in in reviewer quality scores between strategies, χ^2^(3) = 23.93, *p* < 0.001. The effect size was moderate-to-large (Kendall’s W = 0.40), indicating substantial differences in performance across strategies. Figure 2 and Table 4 show visual comparisons of the results.

Post hoc pairwise comparisons using Wilcoxon signed-rank tests with Bonferroni correction showed that Strategy A yielded significantly lower scores than Strategy B (*p* = 0.003), Strategy C (*p* = 0.003), and Strategy D (*p* = 0.025). In contrast, we found no statistically significant differences among Strategies B, C, and D after correction (see Table 5).

### 3.3. Multivariable Analysis

To isolate the relative contributions of case complexity and prompting strategy to expert-rated documentation quality, we employed a multivariable linear regression model. The dependent variable was the median reviewer score (MedianReviewers), derived from two independent expert ratings on a four-point ordinal scale.

The model specification was:

MedianReviewers ~ Case + Strategy.

The model was statistically significant (F = 3.128, *p* = 0.0002853), explaining 54.7% of variance (R^2^ = 0.5469; adjusted R^2^ = 0.3721). SBP demonstrated the largest effect size and strongest statistical significance (see Table 6).

In contrast, most case-level coefficients were non-significant, indicating that prompt architecture explained substantially more variance in quality ratings than case-specific complexity.

### 3.4. Inter-Rater Reliability

Quadratic weighted Cohen’s kappa (see Table 7) indicated fair overall agreement (κ = 0.354; 95% CI 0.15– 0.53). Strategy-level kappas were lower and exhibited wide confidence intervals.

Inter-rater agreement between the two primary reviewers across all reports were stratified by prompting strategy. We used bootstrap resampling (2000 iterations) to calculate confidence intervals.

Given the blinded within-case comparative design, moderate inter-rater agreement does not invalidate relative strategy differences, but it underscores the inherent subjectivity of qualitative documentation assessment.

### 3.5. Exploratory Hallucination Analysis

A total of 1459 phrases were analyzed, of which 269 were classified as titles and excluded. Only clinically relevant phrases were included in the denominator.

The final dataset included 1190 clinically relevant phrases.

Overall, 71.3% (849/1190) of phrases were classified as containing no hallucinations, 19.7% (234/1190) as deductions, and 9.0% (107/1190) as hallucinations.

In the comparison across strategies, we observed substantial variability in hallucination rates across generation strategies, as detailed in Table 8:

- The section-based prompt strategy showed the lowest hallucination rate at the phrase level (1.4%; 7/488).
- The single prompt strategy also demonstrated low hallucination frequency (6.3%; 16/252).
- The section-based prompt with writing refinement strategy showed a higher hallucination rate (11.1%; 17/153).
- Human-authored reports exhibited the highest hallucination proportion (22.6%; 67/297).

At the report level, median hallucination rates were:

- Single prompt strategy: 1.5%.
- Section-based prompt strategy: 1.4%.
- Section-based prompt with writing refinement strategy: 11.1%.
- Human-authored reports: 24.7%.

The section-based prompt architecture yielded the lowest proportion of hallucinated content at the phrase level. However, its performance at the report level was comparable to that of the single prompt strategy, suggesting that both approaches achieved similarly favorable factual consistency in this exploratory setting.

In contrast, increasing segmentation to 20 prompts in the section-based prompt with writing refinement strategy did not improve performance and was associated with a higher proportion of unsupported content. Notably, human-authored reports showed the highest hallucination rate, indicating that unsupported or non-verifiable information is not exclusive to AI-generated outputs.

### 3.6. Qualitative Coding Procedures and Consensus Development

Thematic clustering of reviewer comments identified five recurrent domains: structural coherence, functional specificity, redundancy, reasoning adequacy, and stylistic clarity.

**Figure 3:**
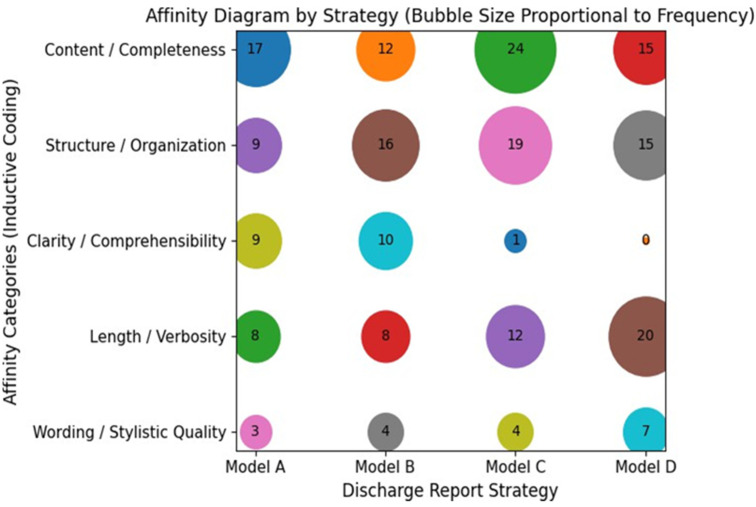
Affinity diagram of qualitative reviewer feedback by strategy. Bubble size is proportional to the frequency of coded observations within each thematic domain. Comments were clustered into five categories: content/completeness, structure/organization, clarity/comprehensibility, length/verbosity, and wording/stylistic quality. Modular section-based prompting concentrated on structural and completeness-related observations, whereas writing refinement prompting was more frequently associated with verbosity-related comments.

Figure 3 shows the distribution of qualitative codes across strategies. Modular prompting (SBP) demonstrated the highest thematic density in structural and completeness-related domains, whereas SBP+W was more frequently associated with verbosity-related observations.

SBP was most consistently associated with improved structural organization and logical sequencing. SP outputs were more variable in coherence, while human-authored reports demonstrated contextual nuance but less structural standardization.

Qualitative findings converged with quantitative results, reinforcing the interpretation that architectural segmentation improves perceived documentation quality.

## 4. Discussion

This study provided controlled empirical evidence that prompt architecture influenced expert-rated documentation quality in complex longitudinal clinical reporting tasks. While prior work has demonstrated the feasibility of AI-generated discharge summaries in acute and primary care settings, few studies systematically isolated prompting architecture as an independent experimental variable in rehabilitation documentation [1,2,3,4,5,6,20].

Across analyses, structured prompting emerged as a key determinant of perceived documentation quality. Section-based approaches consistently achieved higher ratings, suggesting that explicit structural scaffolding, rather than generative capacity alone, played a central role in shaping clinically relevant outputs. These results support the interpretation that predefined structural frameworks may enhance coherence, completeness, and usability of complex clinical documentation. In rehabilitation settings, where longitudinal multidisciplinary documentation is often extensive and heterogeneous, improved structural consistency may additionally facilitate information retrieval, interdisciplinary communication, and continuity of care.

However, these conclusions should be interpreted cautiously. The evaluation relied on subjective expert ratings with moderate inter-rater agreement (κ = 0.354), and the study design did not allow disentangling structural effects from potential stylistic or familiarity biases. Therefore, the observed advantage of structured scaffolding should be understood as a signal of perceived documentation quality rather than objective superiority. This level of agreement indicates that evaluators applied the rating criteria with notable variability, introducing additional uncertainty into the interpretation of perceived quality differences. Consequently, the reliability constraints of the assessment process may have attenuated or inflated observed effects, underscoring the need for more standardized rating frameworks and improved rater calibration in future studies.

In this controlled within-case design, all LLM-based strategies achieved higher expert-rated quality scores than non-standardized human-authored reports. Importantly, because human-authored reports were not generated under a standardized documentation framework, observed differences should not be interpreted as evidence of overall clinical superiority of AI-generated reports. Rather, the comparison primarily reflects differences in perceived structural integrity, coherence, and completeness. Results should therefore be interpreted within this evaluative framework [7,21].

Among prompting strategies, the section-based prompt (SBP) achieved the highest median scores and the largest regression coefficient. However, post hoc pairwise comparisons did not demonstrate statistically significant differences between LLM-based strategies after correction for multiple testing. Accordingly, we interpreted differences among SP, SBP, and SBP+W with caution, particularly given the limited sample size.

Exploratory multivariable model suggested that prompting strategy accounted for more variability in expert ratings than case-level factors. While this observation aligned with the hypothesis that architectural design exerted a measurable influence, the ordinal nature of the dependent variable and the modest sample size constrained interpretability. As such, the model should be considered descriptive and hypothesis-generating.

An important interpretative consideration concerned the comparator. Human-authored reports were not standardized, and observed differences likely reflected the impact of architectural standardization rather than intrinsic superiority of AI-generated content. The findings further supported the potential value of structured documentation frameworks, whether implemented through AI prompting or human-designed templates [22].

Notably, the addition of a writing refinement layer (SBP+W) did not confer incremental benefit, reinforcing the central role of structural organization over post hoc linguistic refinement. This suggested that macro-level segmentation may be a dominant driver of perceived documentation quality in long context settings [11,12,13,14,15,16,23,24,25].

Latest evidence on section-wise generation and retrieval-augmented generation (RAG) pipelines provides additional context for these observations. Section-level generation frameworks have demonstrated improvements in factual consistency and reductions in contextual drift in clinical summarization tasks [26]. Likewise, question-driven RAG approaches for EHR summarization show that grounding generation in retrieved clinical evidence can effectively constrain hallucinations [27]. Although our prompting architecture did not incorporate retrieval mechanisms, the performance of SBP aligns with the broader observation that structural decomposition enhances reliability. Future work could integrate RAG components to further strengthen factual grounding.

This study had several limitations, including modest sample size, moderate inter-rater agreement, single-model evaluation (GPT-5.2), retrospective design, and the absence of objective factual error auditing or safety outcome assessment. Although a limited hallucination analysis was performed, it remained exploratory due to the small number of reports evaluated and should not be considered sufficient to establish the factual reliability or clinical safety of the generated documentation. Recent work from Pal and colleagues, introduced the Med-HALT benchmark for systematically evaluating hallucinations in the medical domain. It highlights the growing need for structured, domain-specific hallucination assessment frameworks. The absence of such standardized hallucination stress-tests in our study represents an additional limitation and underscores the importance of incorporating validated medical hallucination benchmarks in future research [28]. Generalizability beyond inpatient rehabilitation remains uncertain. Overall, structured prompt design may represent a modifiable and clinically relevant factor in AI-assisted documentation. However, findings should be considered preliminary and hypothesis-generating, warranting further validation in larger, methodologically robust studies incorporating objective quality and safety metrics. Collectively, these findings suggest that prompt architecture itself may represent an underrecognized determinant of documentation reliability in long context clinical generation tasks.

## 5. Conclusions

In the context of inpatient rehabilitation discharge documentation, using a structured scaffolding in an AI prompt architecture positively influenced expert-rated documentation quality.

Within this dataset, the prompting strategy accounted for a larger share of explainable variability in expert ratings than case-level variability, suggesting that architectural prompt design may represent a controllable implementation variable in clinical LLM deployment.

From a bioengineering perspective, prompt architecture may function as a system-level design intervention that shapes the interaction between generative models and clinical documentation workflows. Structured modular prompting appears to offer a promising and scalable strategy for enhancing perceived documentation reliability in complex longitudinal reporting tasks.

## Figures and Tables

**Table 1:** Summary of the main characteristics of the evaluated prompt architectures.

Parameter	Unified Architecture	Segmented Architecture	Segmented + Refinement
Processing strategy	Single-step inference	Section-based processing	Two-stage per section
Number of prompts	1	10	20
Attention distribution	Global	Local	Segregated
Traceability	Implicit (uncertainty detection)	Reference-based validation	Deterministic source mapping
Hallucination control	Constraint-based	Validation loops	Structural + source-linked
Computational complexity	Low	Moderate	High
Main limitation	Cognitive overload	Cross-section coherence	Reduced flexibility

**Table 2:** Application of the Prompt Canvas framework in the design of the prompt architectures.

Element	Definition	Implementation
Role	Defines model expertise	Specialist in Physical and Rehabilitation Medicine
Audience	Target end-user	Continuity-of-care physician
Context	Input characteristics	Longitudinal inpatient clinical records
Task	Objective	Generation of structured discharge reports
Process	Execution logic	Architecture-dependent workflows
Anchors	Safety constraints	Exclusion of irrelevant or unsafe data
Format	Output structure	Section-based or schema-driven outputs
Style	Language constraints	Concise and objective clinical language
References	Data grounding	Strict use of source documentation
Evaluation	Quality control	Internal validation and cross-checking

**Table 3:** Descriptive statistics of ratings across strategies.

Strategy	Mean	Median	SD	IQR
A	1.65	2.00	0.69	1.00
B	2.33	2.00	0.57	1.00
C	2.49	3.00	0.63	1.00
D	2.14	2.00	0.71	1.00

SD: standard deviation; IQR: interquartile range.

**Table 4:** Comparison of reviewer quality scores across strategies.

Strategy	Median (IQR)
A	1.83 (1.38–2.00)
B	2.42 (2.00–2.50)
C	2.50 (2.00–3.00)
D	2.00 (2.00–2.50)

Friedman test: χ^2^(3) = 23.93, *p* = 2.59 × 10^−5^. Kendall’s W = 0.399.

**Table 5:** Post hoc Wilcoxon signed-rank test with Bonferroni correction.

Comparison	*p*
A vs. B	0.00346
A vs. C	0.00255
A vs. D	0.02455
B vs. C	0.84722
B vs. D	0.98388
C vs. D	0.10482

**Table 6:** Multivariable linear regression model evaluating the effect of case complexity and prompting strategy on expert ratings.

Predictor	Estimate	Std. Error	*p*-Value
B (SP)	+0.675	0.149	<0.001
C (SBP)	+0.850	0.149	<0.001
D (SBP+W)	+0.500	0.149	0.001

Model statistics: R^2^ = 0.5469; adjusted R^2^ = 0.3721; F(22,57) = 3.128; *p* = 0.0002853.

**Table 7:** Quadratic weighted Cohen’s kappa (κ) for inter-rater reliability across strategies.

Strategy	κ	95% CI	n
Overall	0.354	0.15–0.53	80
A (human)	0.322	−0.08–0.62	20
B (SP)	0.250	−0.12–0.56	20
C (SBP)	0.097	−0.14–0.50	20
D (SBP+W)	0.095	−0.25–0.46	20

κ, Cohen’s kappa coefficient; CI, confidence interval; SP, single prompt; SBP, section-based prompt; SBP+W, section-based prompt with writing refinement.

**Table 8:** Summary of the exploratory phrase-level hallucination analysis across report generation strategies.

Strategy	Audited Reports (n)	Clinical Phrases (n)	No Hallucination n (%)	Deduction n (%)	Hallucination n (%)	Median Report-Level Hallucination Rate (%)
Human-authored	2	297	197 (66.3%)	33 (11.1%)	67 (22.6%)	24.7%
Single prompt	3	252	181 (71.8%)	55 (21.8%)	16 (6.3%)	1.4%
SBP	3	488	358 (73.4%)	123 (25.2%)	7 (1.4%)	1.5%
SBP+W	2	153	113 (73.9%)	23 (15.0%)	17 (11.1%)	11.1%
Total	10	1190	849 (71.3%)	234 (19.7%)	107 (9.0%)	—

## Data Availability

The data presented in this study are available on request from the corresponding author due to privacy restrictions.

## Abbreviations

The following abbreviations are used in this manuscript:

SD	Standard Deviation
IQR	Median with Interquartile Range
CEIm	Comité de Ética de la Investigación con Medicamentos
LLM	Large Language Model
SP	Single Prompt
SBP	Section-Based Prompt
SBP+W	Section-Based Prompt with Writing Refinement
WHO	World Health Organization

## Appendix A

### Expert Evaluation Rubric

How would you rate the report you just downloaded?

- Poor [0]—The report is incomplete, with serious problems in comprehension, structure, or accuracy. Major errors and insufficient supporting data limit its reliability.
- Fair [1]—The report shows issues with coherence, lacks depth, or contains minor content errors. The writing may be unclear in some areas, affecting readability.
- Good [2]—The report meets the essential requirements. It has a clear structure, reasonable arguments, and appropriate use of information. There may be minor inaccuracies or areas for improvement, but they do not hinder understanding.
- Excellent [3]—The report is very well structured, coherent, complete, and tailored to the target audience. The content is accurate, well-supported, and backed by appropriate data and evidence. The writing is fluent and free of significant errors.

## Appendix B. Representative Prompt Specifications and Operational Constraints

### Appendix B.1. Unified Instruction Architecture (Single Prompt, SP)

The unified architecture processed the complete anonymized clinical record and all generation instructions within a single inference step. The model was assigned a fixed expert role and instructed to generate a complete rehabilitation discharge report using only the information available in the source documentation.

Representative operational instructions included:

- “You are a physician specializing in Physical and Rehabilitation Medicine and an expert medical assistant in the synthesis of clinical documents.”
- “Use only the information provided in the source documentation.”
- “Do not invent findings, doses, dates, or treatments.”
- “Prioritize the most recent information when chronological conflicts arise.”
- “If required data are missing, include a section entitled ‘Required missing data’.”

The internal workflow was organized into four sequential stages:

1. Initial analysis of longitudinal progress notes.
2. Extraction of relevant clinical milestones.
3. Narrative synthesis and report generation.
4. Internal validation and consistency checking.

The generated output followed a predefined rehabilitation discharge report structure, including admission data, clinical evolution, physical examination, treatment at discharge, diagnoses, and continuity-of-care recommendations.

### Appendix B.2. Segmented Instruction Architecture (Section-Based Prompting, SBP)

The segmented architecture decomposed the discharge report into ten predefined clinical sections:

1. Reason for admission.
2. Past medical history.
3. Baseline functional status.
4. Current illness.
5. Physical examination.
6. Clinical course.
7. Complementary tests.
8. Functional scales.
9. Diagnoses.
10. Management and follow-up plan.

Each section was generated independently using a dedicated prompt. The workflow for each section contained two sequential internal phases:

1. Extraction of clinically relevant information from the source notes.
2. Structured narrative generation for the target subsection.

Representative validation instructions included:

- “Cross-check each recommendation against its reference; if it does not match or the reference does not exist, remove that recommendation.”
- “If the information corresponds to treatment received only during admission and not continued after discharge, exclude it.”
- “Maintain chronological consistency across all statements.”

This architecture aimed to reduce contextual overload by restricting the model’s attention window to a single clinical domain at a time.

### Appendix B.3. Segmented Architecture with Sequential Refinement (SBP+W)

The refinement architecture separated extraction and narrative generation into two isolated computational stages for each report section.

**Stage 1: Structured Extraction**

The model extracted clinical variables into structured schema-based outputs without generating free narrative text.

Representative extraction schema:

</> JSON

{

“field”: “diagnosis”,

“value”: “Right hemispheric ischemic stroke”,

“sources”: [

{

“text_snippet”: “Patient admitted after right MCA ischemic stroke…”

}

]

}

Representative extraction constraints included:

- “Return exclusively structured information.”
- “Do not generate narrative interpretation.”
- “Link every extracted variable to the corresponding source fragment.”

**Stage 2: Narrative Refinement**

The structured outputs were subsequently transformed into clinically readable discharge report sections.

Representative refinement instructions included:

- “Preserve factual fidelity.”
- “Do not introduce novel clinical information.”
- “Avoid dangling commas, empty connectors, and phrases such as ‘not recorded’.”
- “Maintain concise and objective medical language.”

This architecture prioritized deterministic traceability and hallucination mitigation through explicit separation between factual extraction and linguistic refinement.

### Appendix B.4. Standardized Constraints Across All Prompting Strategies

The following operational constraints remained constant across all experimental conditions:

- Fixed expert role assignment.
- Exclusive use of anonymized source documentation.
- Prohibition of unsupported information generation.
- Chronological prioritization of recent clinical data.
- Structured discharge report organization.
- Formal clinical language requirements.
- Independent processing of each clinical case.
- No iterative prompt optimization after protocol finalization.

All prompting strategies were executed under identical model conditions using GPT-5.2, operating in standard reasoning mode with default parameters and consistent generation settings throughout the study.
